# Trojan Horses: Conjugating Siderophores and Antibiotics—A New Approach to Treating *Pseudomonas aeruginosa* Infection

**DOI:** 10.3390/microorganisms14040891

**Published:** 2026-04-16

**Authors:** Wei Xiao, Xin Ma, Dandan Liu, Shengli Li, Juanli Cheng, Jinshui Lin

**Affiliations:** 1 Shaanxi Key Laboratory of Research and Utilization of Resource Plants on the Loess Plateau, College of Life Sciences, Yan’an University, Yan’an 716000, China; 18792388186@163.com (W.X.); 14791685086@163.com (X.M.); 17829553016@163.com (D.L.); 18181017935@163.com (S.L.); 2 Research Center of Avian Disease, College of Veterinary Medicine, Sichuan Agricultural University, Chengdu 611130, China

**Keywords:** *Pseudomonas aeruginosa*, siderophores, pyoverdine, pyochelin, Trojan horse strategy

## Abstract

*Pseudomonas aeruginosa* is a common Gram-negative bacterium in hospital infections and one of the main pathogens causing opportunistic infections in humans. In recent years, the drug resistance of *P. aeruginosa* has become increasingly severe. Therefore, it is urgent to explore new targets for antibacterial therapy. In *P. aeruginosa*, iron is an essential element not only for cell growth but also for successful infection. Two siderophores are produced by *P. aeruginosa*: pyoverdine and pyochelin. They help *P. aeruginosa* to obtain iron and play an important role in interspecific competition, anti-oxidative stress, and virulence. Furthermore, siderophores have been used to design “Trojan horse” antibiotics. These antibiotic–siderophore conjugates enter the cytoplasm of *P. aeruginosa* via siderophore uptake systems for pyoverdine and pyochelin, releasing antibacterial substances and exerting corresponding effects against *P. aeruginosa*. This review discusses the synthesis, secretion, and uptake of siderophores in *P. aeruginosa* as well as the role of the “Trojan horse” strategy in treating *P. aeruginosa* infections.

## 1. Introduction

*Pseudomonas aeruginosa* (*P. aeruginosa*) is a Gram-negative bacterium and a widespread, opportunistic pathogen. Immunocompromised patients, such as patients with cystic fibrosis, human immunodeficiency virus 1, cancer, burns, or a history of surgery, are susceptible to *P. aeruginosa* infection [1,2,3,4]. *P. aeruginosa* can quickly adapt to the environment and develop resistance to antibiotics, and thus, effective treatments and control strategies are lacking in clinical practice. As such, *P. aeruginosa* is widely recognized as one of the most threatening pathogens to human health. Owing to its high propensity to develop antibiotic resistance and cause severe clinical infections, this bacterium has been consistently classified as a top-priority pathogen [5,6,7]. Vaccines against *P. aeruginosa* are still being developed. Although some vaccines against *P. aeruginosa* have shown good results in animal tests, they have failed in clinical trials [1]. Therefore, it is particularly important to develop new antibacterial strategies against *P. aeruginosa.*

Iron is an essential element in all organisms and is a cofactor for many enzymes involved in key metabolic processes, such as cellular respiration, nucleotide biosynthesis, DNA replication, transcription, and repair [8,9,10]. Although the earth is rich in iron, it is not easy to obtain, resulting in its low bioavailability [11]. Under aerobic conditions, iron is oxidized into insoluble Fe^3+^-containing compounds, decreasing free iron content [12]. Conversely, Fe^2+^ is abundant under anaerobic conditions or at low pH [12]. This makes it difficult for aerobic bacteria to obtain iron directly from the environment. Thus, bacteria have evolved different strategies to obtain iron, such as via extracellular Fe^3+^-chelating molecules (produced by themselves or other microorganisms) [13]. These Fe^3+^-chelating molecules, previously known as siderochromes, sideramines, and sideromycins, are now generally referred to as siderophores [14]. To date, more than 500 different siderophores have been identified [15]. According to the mode of complexation with Fe^3+^, siderophores are roughly classified as phenolate, catecholate, hydroxamate, hydroxycarboxylate, or mixed types [16]. When a siderophore binds Fe^3+^ to form a complex, it is recognized by specific TonB-dependent receptors for cell uptake [15].

Iron also plays an important role in *P. aeruginosa* survival and infection. Upon *P. aeruginosa* infection, the host responds to it via, for example, “nutritional immunity” [17]. In response, *P. aeruginosa* secretes two siderophores with high iron affinity: pyoverdine (PVD) and pyochelin (PCH) [18]. During infection, *P. aeruginosa* obtains most of its iron from the host, using the heme from hemoglobin and its siderophores, PVD and PCH, to chelate Fe^3+^ from transferrin and lactoferrin. Among these pathways, siderophore-mediated iron uptake dominates in acute infection, whereas heme serves as the primary iron source during chronic infection [18,19,20]. The battle for iron is an important process in the host–pathogen interaction, and the effectiveness of a pathogen to acquire this metal can determine the success or failure of an infection [11]. Therefore, understanding the mechanism of iron acquisition is a promising approach to combating *P. aeruginosa* infection. Moreover, the active uptake behavior of *P. aeruginosa* toward siderophores has been utilized to develop new types of antimicrobial substances. In recent years, an antibacterial concept called the “Trojan horse” strategy has been proposed. This strategy chemically conjugates antimicrobial active substances with *P. aeruginosa* siderophores and their analogues to form siderophore–antibiotic complexes. In low-iron environments, *P. aeruginosa* actively absorbs Fe^3+^ through a siderophore-mediated iron uptake system. To this end, these complexes exploit the specific recognition systems and efficient transport abilities of *P. aeruginosa* for internalization, after which they inhibit the bacteria. For this reason, the complex is called the “Trojan horse” antibiotic [14,21,22,23]. To this end, siderophores exhibit enormous applicative potential in the design and development of targeted antibacterial drugs that use the “Trojan horse” strategy against *P. aeruginosa*. This article reviews the synthesis, secretion, and iron uptake mechanisms of siderophores in *P*. *aeruginosa* as well as the application of siderophore-based “Trojan horse” strategies in treating *P. aeruginosa* infections.

## 2. Pyoverdine (PVD) and Pyochelin (PCH) in *P. aeruginosa*

Pathogens face the problem of decreased iron availability during infection because iron in the host is chelated in heme molecules or other iron transporter proteins [24]. To this end, *P. aeruginosa* has evolved high-affinity siderophores (PVD and PCH) with uptake pathways. These siderophores chelate extracellular Fe^3+^ to form stable complexes, which are then transported into the cell via PVD- and PCH-uptake pathways [18,25].

### 2.1. PVD Biosynthesis, Secretion, and Uptake

**Figure 1:**
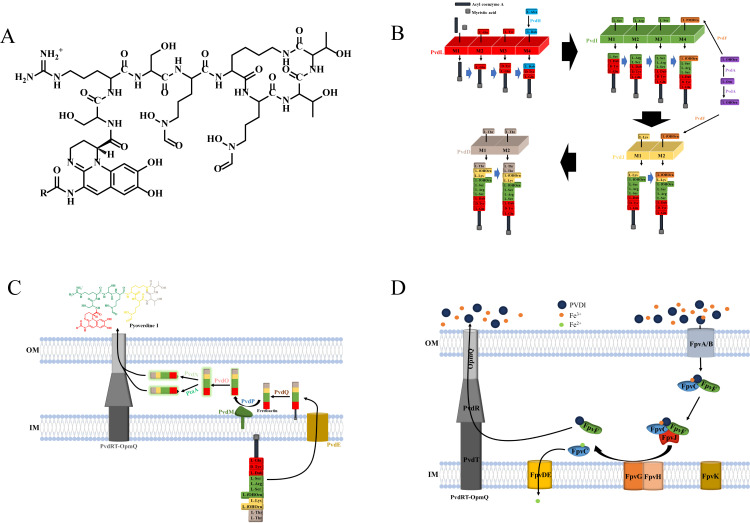
Pyoverdine-1 (PVDI) structure and pathways in *P. aeruginosa*. (**A**) PVDI structure. A mixed-type siderophore containing three Fe^3+^-chelating functional groups: a catecholate-like dihydroxyquinoline chromophore, a hydroxamate group, and a hydroxycarboxylate group. (**B**) Biosynthesis. The peptide backbone of PVDI is assembled by non-ribosomal peptide synthetases (NRPSs) including PvdL, PvdI, PvdJ, and PvdD. (**C**) Secretion. The immature PVDI precursor is transported into the periplasm via the PvdE transporter. In the periplasm, its chromophore is sequentially matured by the enzymes PvdN, PvdO, PvdP, and PvdQ. Fully matured PVDI is then secreted into the extracellular space through the PvdRT-OpmQ efflux pump to chelate environmental Fe^3+^. (**D**) Uptake. Extracellular Fe^3+^-bound PVDI (ferric-PVDI) is recognized by the outer membrane receptors FpvA/FpvB and transported into the periplasm. The complex is then delivered to the inner membrane reductase FpvG mediated by FpvF–FpvC, where Fe^3+^ is released from PVDI and reduced to Fe^2+^. During this process, FpvJ forms a stable complex with FpvC and FpvF, and promotes efficient Fe^3+^ reduction and Fe^2+^ transfer via interactions with FpvG and FpvH. The resulting Fe^2+^ binds to FpvC and is subsequently transported into the cytoplasm by the inner membrane ABC transporter FpvDE for bacterial utilization, whereas apo-PVDI binds to FpvF and is recycled back to the extracellular environment via the PvdRT-OpmQ efflux system.

PVD produced by *P. aeruginosa* is a complex siderophore composed of a polypeptide containing 11 amino acids, a chromophore group, and a myristic or myristoleic acid chain at its N-terminal end [26,27,28,29] (Figure 1A).

*P. aeruginosa* can produce four different PVDs: PVDI–IV [29,30]. PVDI is derived from *P. aeruginosa* PAO1 [31] and is representative of PVDs in general. PVDI synthesis begins in the cytoplasm, after which it is processed and matured in the periplasm, and finally secreted into the extracellular space via the PvdRT-OpmQ efflux pump. For uptake, PVDI–Fe^3+^ is internalized by the specific outer membrane transporter FpvA or FpvB, entering through a unique pathway [32].

#### 2.1.1. Biosynthesis of PVD

The biosynthesis of PVDI in *P. aeruginosa* is a multi-step process, which is mainly divided into two stages. First, a periplasmic peptide precursor form designated as ferribactin (a yellowish non-fluorescent compound) is synthesized in the cytoplasm [32]. It contains 11 amino acids with the following sequence: L-Glu-D-Tyr-L-Dab-D-Ser-L-Arg-D-Ser-L-fOHOrn-L-Lys-L-fOHOrn-L-Thr-L-Thr, of which L-Dab (L-2,4-diaminobutyrate) and L-fOHOrn (L-N_5_-formyl-N_5_-hydroxyornithine) are two uncommon amino acids [29,31].

Second, ferribactin is processed in the periplasm to form a siderophore [31,32] (Figure 1B). The biosynthesis of ferribactin involves four cytosolic non-ribosomal polypeptide synthases (PvdL, PvdI, PvdJ, and PvdD), three cytosolic enzymes (PvdH, PvdA, and PvdF), and several enzymes that modify PVDI [11,29,31]. Both the chromophore backbone and peptides are synthesized from non-ribosomal polypeptide synthases, which are large enzymes that contain multiple modules. Their functions are summarized as activating amino acids, which can be assembled into peptide chains. A single module of non-ribosomal polypeptide synthases can only activate and modify a specific amino acid. As such, the sequence and number of non-ribosomal polypeptide synthases and their modules determine the polypeptide chain sequence [32,33].

The first synthase to assemble the PVDI precursor is PvdL, which contains four modules [34]. The first module of PvdL contains an acyl coenzyme A ligase domain that catalyzes the acylation of fatty acids (myristic or myristoleic) [29,31,32,34]. This acylation occurs to maintain the peptide precursor at the membrane, promote the assembly of PVDI, and prevent PVDI from chelating iron and other metal ions [29,31,35,36].

The second module of PvdL catalyzes the bond between L-Glu and myristic-CoA or myristoleic-CoA formed in the first module [29,31,32]. The third module of PvdL incorporates D-Tyr [29]. It contains an epimerization domain that isomerizes L-Tyr to D-Tyr [29]. The fourth module incorporates L-Dab to form a tetrahydropyrimidine ring that is the precursor to the dihydroxyquinoline chromophore [29,31,32]. PvdI also has four modules, and the second and fourth modules contain epimerization domains [29,37]. PvdI connects L-Ser, L-Arg, L-Ser, and L-hfOrn to L-Glu-D-Tyr-L-Dab in turn, and forms L-Glu-D-Tyr-L-Dab-D-Ser-L-Arg-D-Ser-L-fOHOrn [29,31,37]. PvdJ and PvdD are two-module non-ribosomal polypeptide synthases; PvdJ connects L-Lys and L-fOHOrn in turn with the peptide formed by PvdI, and PvdD subsequently connects two L-Thr with L-Glu-D-Tyr-L-Dab-D-Ser-L-Arg-D-Ser-L-fOHOrn [29,31].

In addition, PvdH, PvdA, and PvdF promote the assembly of ferribactin. PvdH is an aminotransferase with transaminase activity, which can convert L-Asp into L-Dab [38]. PvdA is an L-ornithine N_5_-monooxygenase, which catalyzes the hydroxylation of L-Orn to L-OHOrn [29,39,40]. L-OHOrn is then methylated to L-fOHOrn with the help of PvdF and participates in the biosynthesis of ferribactin [29,39,40]. The products catalyzed by PvdA and PvdF are only involved in the biosynthesis of PVDI [31,41].

At this point, ferribactin biosynthesis is completed; however, the chromophore of the PVDI precursor is still immature and needs further processing to produce fluorescence [36,42,43]. The uptake pathway of PVD is illustrated in Figure 1C. Ferribactin is exported across the inner membrane by the PvdE ABC transporter to the periplasm [32,36]. PvdN, PvdO, PvdP, and PvdQ are involved in chromophore maturation. The PvdQ acylase enzyme from the N-terminal nucleophile (NTN) hydrolase family detaches the fatty acid chain, yielding ferribactin [32,35,43,44,45].

Subsequently, ferribactin binds to tyrosinase PvdP with the help of the periplasmic protein PvdM (dipeptidase) [46]. PvdP catalyzes the conversion of ferribactin into PVDI in three steps. (i) The D-Tyr residue within the ferribactin molecule is specifically hydroxylated by PvdP to yield a catechol moiety. (ii) PvdP recognizes the peptide backbone in the chromophore precursor region of ferribactin and catalyzes intramolecular cyclization; this process forms the third heterocyclic ring and generates the mature PVDI chromophore. (iii) The catechol moiety that provides the third iron chelation site for PVD is restored to its active form [17,38]. Afterward, PvdO finalizes chromophore synthesis, using non-fluorescent PVDI as the substrate. It catalyzes oxidative dehydrogenation of the chromophore core to extend the conjugated double bond system, thereby converting non-fluorescent PVDI into fluorescent PVDI [33,47,48,49,50].

Finally, through side-chain modification pathways, the side chain of the N-terminal glutamic acid (Glu) residue in fluorescent PVDI is converted into two distinct products: succinamide (catalyzed by PvdN) or α-ketoglutarate (formed by PtaA) [28,51]. This modification can transform PVDI into different variants, such as α-ketoglutaric acid or succinimide-derived variants, which increases PVDI adaptability to different environmental conditions [28,29,51]. After biosynthesis, PVDI is stored in the periplasm [31,32], from which it can be secreted into the extracellular space by the ATP-dependent efflux pump PvdRT-OpmQ [26,29,31,32]. This pump is distributed across all parts of bacteria [52], resulting in PVDI excretion all around bacteria [31].

#### 2.1.2. Uptake and Secretion of PVD

In the extracellular environment, PVD chelates Fe^3+^ to form the PVD–Fe^3+^ complex [53], which is recognized by bacterial outer membrane receptors and translocated into the periplasm through the TonB-dependent transporters FpvA and FpvB [26,54,55,56]. The PVD uptake pathway is illustrated in Figure 1D. The PVD–Fe^3+^ complex is then bound by the periplasmic protein complex FpvF–FpvC [57]. Subsequently, FpvF–FpvC transports PVD–Fe^3+^ to the inner membrane reductase FpvG for reduction and dissociation. The formed PVD binds to FpvF, whereas the generated Fe^2+^ is sequestered by FpvC [26,58]. Afterward, FpvF transports PVD to the efflux pump PvdRT-OpmQ to circulate to extracellular re-chelating Fe^3+^ [26,59,60], and FpvC transports Fe^2+^ to the inner membrane ABC transporter FpvDE. Finally, Fe^2+^ is transported into the cytoplasm by FpvDE [57]. In addition to the above proteins, FpvH and FpvJ may also be involved in the transport and dissociation of PVD–Fe^3+^, as FpvH was shown to interact with the inner membrane protein FpvG [26,58]. FpvJ can form a complex with two periplasmic proteins (FpvC and FpvF) [26,58]. These interactions (between the FpvJ–FpvC–FpvF complex and FpvG/FpvH) are proposed to facilitate the efficient reduction of Fe^3+^ or the transfer of Fe^2+^ to FpvDE, thereby facilitating Fe^2+^ translocation into the bacterial cytoplasm.

Although the basic functions of FpvABCDEFG are clear, the protein functions or interaction networks of some genes involved in uptake and secretion (including some of the above genes) are not known. In addition, *fpvWXYZ* may also be involved in iron uptake, as the inactivation of any one of *fpvW*, *fpvC*, *fpvD*, *fpvE*, and *fpvF* can lead to increased expression of the operon *fpvWXYZCDE* (*PA2403-2409*). However, the exact function of *fpvWXYZ* is still unclear [61].

### 2.2. PCH Biosynthesis, Secretion, and Uptake

#### 2.2.1. Biosynthesis of PCH

**Figure 2:**
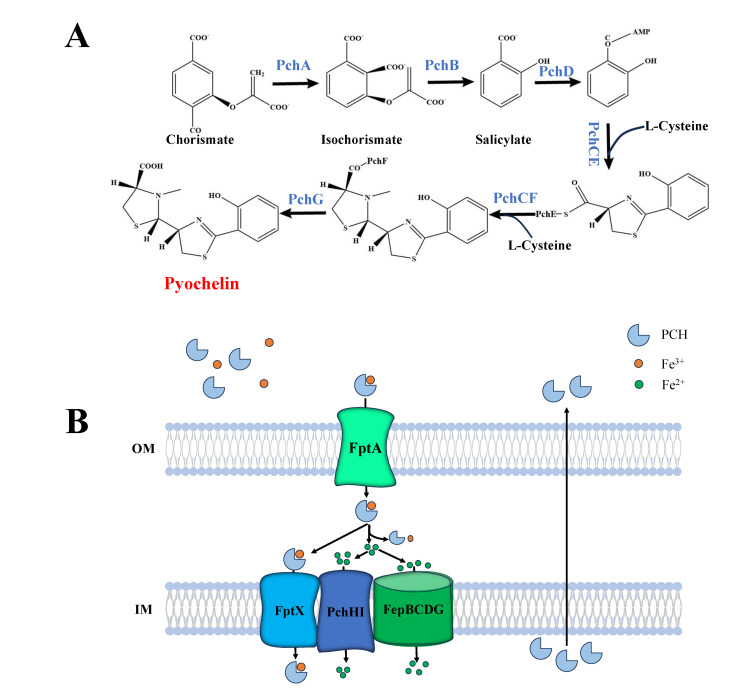
Pyochelin (PCH) in *P. aeruginosa*. (**A**) Biosynthesis. Starting from chorismate, isochorismate is produced under the catalysis of PchA and then converted to salicylate by PchB. Salicylate is activated to salicyl-AMP by PchD, and subsequently condensed with L-cysteine under the joint catalysis of PchC and PchE to form a thiazole ring-containing intermediate. This intermediate further incorporates another molecule of L-cysteine under the catalysis of PchC and PchF to complete the assembly of the second thiazole ring, forming the core scaffold of PCH. Finally, the scaffold is modified by PchG to generate PCH. (**B**) Uptake. Extracellular Fe^3+^-bound PCH (ferric-PCH complex) is recognized by the outer membrane receptor FptA and transported into the periplasm. The complex can transport iron into the cytoplasm via two pathways: first, the intact PCH-iron complex is directly transported into the cytoplasm by FptX; second, Fe^3+^ is reduced to Fe^2+^ in the periplasm and released from PCH, and the free Fe^2+^ is transported into the cytoplasm for bacterial utilization through the inner membrane ABC transporters PchHI and FepBCDG.

PCH is a secondary siderophore produced by *P. aeruginosa* for iron uptake, and its affinity to Fe^3+^ is lower than that of the primary siderophore PVD [29,31]. The structure and biosynthesis pathway of PCH are illustrated in Figure 2A. The biosynthesis of PCH involves seven cytoplasmic enzymes but not periplasmic processing maturation [31,62]. As such, its biosynthesis is simpler than that of PVD. PCH is a condensation product of salicylate and two cysteine molecules, i.e., 2-(2-o-hydroxyphenyl-2-thiazolin-4-yl)-3-methylthiazolidine-4-carboxylic acid [31,63,64].

The biosynthesis of PCH is initiated by PchA, an isochorismate synthase [29]. First, chorismate is converted into isochorismate by PchA [65,66,67], and then isochorismate is converted into salicylate by the isochorismate pyruvate lyase PchB [67,68,69]. Subsequently, salicylate is activated by PchD and PchE, a full-length non-ribosomal polypeptide synthase enzyme harboring an N-terminal P-domain [29,31,32]. PchE contains a cysteine activation domain, which can activate and connect L-Cys to salicylate to form Dha (dehydroalanine). This process requires PchC, which, however, is not necessary for Dha release from PchE [29,31,67,70]. PchF also contains a cysteine domain, which activates new L-Cys. Subsequently, L-Cys is connected and cyclized with Dha by PchF [31,67,68,70,71,72]. The product is released by the reductase PchG [73].

#### 2.2.2. Uptake and Secretion of PCH

The uptake pathway of PCH is illustrated in Figure 2B. When PCH chelates Fe^3+^, the PCH–Fe^3+^ complex is recognized and transported into the periplasm by the outer membrane transporter FptA of *P. aeruginosa*, which is similar to the uptake of PVD–Fe^3+^ by FpvA [63]. Subsequently, PCH–Fe^3+^ is transported into the cytoplasm through the inner membrane transporter FptX. Roche et al. showed that PchH and PchI do not affect PCH production and secretion in *P. aeruginosa* [74]. Instead, PchHI only acts as an ABC transporter, facilitating the movement of Fe^3+^ into the cytoplasm [74]. It is worth noting that the phenotype resulting from the deletion of both the PchHI and FptX transporters is identical to that of FptX deletion alone, indicating that PchHI plays a secondary role in PCH uptake [74]. In addition, this study found that the PchHI complex interacts with FptX [74]. These results suggest that, after PCH–Fe^3+^ enters the periplasm, it mainly enters the cytoplasm through FptX. However, it undergoes dissociation through an as-yet-to-be-identified mechanism, and liberated free Fe^3+^ ions are subsequently translocated into the cytoplasm by PchHI [74].

Our recent report provides evidence of a third inner membrane channel in the PCH pathway in *P. aeruginosa* [75] (Figure 2). FepB, FepC, FepD, and FepG are encoded by *fepB*, *fepC*, *fepD*, and *fepG*, which can form the ABC family inner membrane transport protein complex FepBCDG. After dissociation of PCH–Fe^3+^ in the periplasm, free iron can be transported into the cytoplasm through FepBCDG. Interestingly, FepBCDG associates with both PchHI and FptX, indicating that FepBCDG, PchHI, and FptX may form a larger complex and mediate the transport of Fe^3+^ in the PCH pathway.

The two genes *pchH* and *pchI* located downstream of *pchEF* seem to have the function of an ABC transporter [31]. However, Reimmann et al. found that *pchH* and *pchI* mutations did not affect the production of salicylic acid, Dha, or PCH, indicating that PchHI is not required for PCH biosynthesis or secretion [73]. At present, no studies have reported the secretion mechanism of PCH.

## 3. Siderophore–Antibiotic Coupling

**Figure 3:**
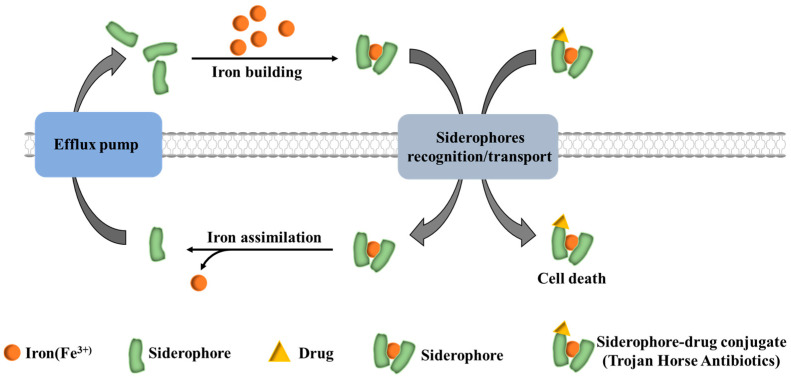
Siderophore-mediated exploitation by “Trojan horses”. Siderophores (green) chelate extracellular Fe^3+^ (orange) to form ferric-siderophore complexes, which are recognized and transported into the cell via the siderophore recognition/transport system. Ferric-siderophore complexes deliver Fe^3+^ into the cell for bacterial iron assimilation, and free siderophores can be recycled back to the extracellular space. When siderophores are conjugated with antibiotics (yellow triangles) to form siderophore–antibiotic conjugates (“Trojan horse” antibiotics), bacteria misrecognize them as natural siderophores and take them up actively, allowing the antibiotics to enter cells and exert their effects, ultimately leading to bacterial death.

Siderophores show much promise for combating drug-resistant microorganisms. As mentioned earlier, the “Trojan horse” strategy leverages specific siderophore recognition and high-efficiency transport systems in bacteria. Antibiotic–siderophore conjugates are actively internalized into bacterial cells, thereby inhibiting and eliminating pathogenic strains [14,21,22] (Figure 3). Notably, mixed-type siderophores—defined by the integration of multiple distinct functional groups within a single molecule—are particularly valuable for this strategy [76]. PVD and PCH, the primary siderophores produced by *P. aeruginosa*, are both representative mixed-type siderophores. PVD possesses three major Fe^3+^ chelating groups: catecholate, hydroxamate, and hydroxycarboxylate, while PCH features a salicylate-derived catechol-like motif and a thiazoline ring scaffold, enabling efficient Fe^3+^ coordination through multiple ligand sites [32].

These two siderophores also have structurally or functionally similar analogs in other microorganisms. PVD, as a conserved member of the fluorescent siderophore family in fluorescent pseudomonads, possesses homologous analogs across a variety of fluorescent *Pseudomonas* species [77,78]. In contrast, the thiazoline-containing hybrid scaffold of PCH also has structurally similar siderophores in other pathogenic bacteria, such as *Burkholderia* and *Yersinia*, which share similar iron-chelating mechanisms and biological functions [79,80].

The abundant chelating groups in PVD, PCH, and their analogues provide ideal sites for conjugation with antibacterial agents. Therefore, these siderophores exhibit significant application prospects in the design of “Trojan horse” antibiotics.

### 3.1. PVD–Antibiotic Coupling

PVD can be successfully designed as a “Trojan horse” antibiotic because it contains groups that chelate Fe^3+^, which causes bacteria to mistakenly internalize it due to normal growth needs.

#### 3.1.1. Conjugates of Penicillin Antibiotics and Siderophores

**Figure 4:**
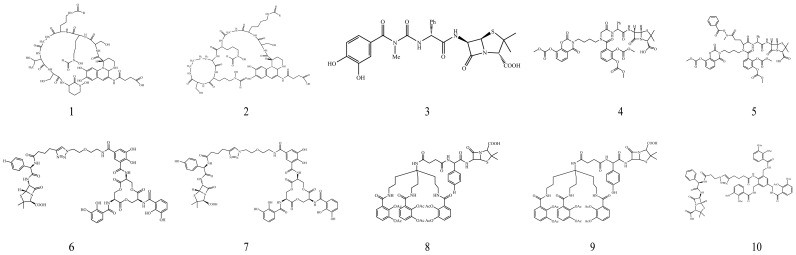
Conjugates of penicillin antibiotics and siderophores.

Penicillin antibiotics inhibit transpeptidase activity by irreversibly binding to penicillin-binding proteins on the bacterial cell wall. This blocks peptidoglycan synthesis in the bacterial cell wall, which impairs bacterial cell wall synthesis and causes death under osmotic pressure [81]. As shown in Figure 4, in 1998, Kinzel et al. synthesized conjugate 1 of PVDI with ampicillin and conjugate 2 of PVDII with ampicillin. Both contain dihydroxy acid as the connecting group between the Lys residues of PVDI and PVDII and ampicillin, as the ten-carbon structure of dihydroxy acid helps maintain the configuration of the coupling and recognition of the conjugate by the outer bacterial membrane. The minimum inhibitory concentration (MIC) values of conjugates 1 and 2 were 0.39 and 0.24 μm/L, respectively, indicating that they could enter *P. aeruginosa* through iron uptake [82]. In the 1920s, Ohi et al. synthesized a new ureidopenicillin derivative containing a monocatechol group: conjugate 3. It showed strong antibacterial activity against *P. aeruginosa*, with a more than 30-fold lower MIC value against *P. aeruginosa* than that of penicillin [83]. Additionally, Heinisch’s team synthesized a siderophore of the biscatechol group based on N-aminoalkylglycine, N-aminopropyl-alanine, and N-aminopropyl-4-aminopentanoic acid, and linked it to ampicillin to form conjugate 4. The bacteriostatic activity of conjugate 4 against *P. aeruginosa* (MIC < 0.05 μg/mL) was approximately 1000-fold higher than that of ampicillin (MIC > 100 μg/mL) [84]. They further studied siderophores of the biscatechol group and coupled the synthesized catechol derivatives with β-lactam antibiotics. The new conjugate 5 (based on 5 <aminoethyl>-2,5,8-triazaalkylbenzoic acid) can play a role in the iron transport system of bacteria. It also has strong antibacterial activity against both Gram-negative and Gram-positive bacteria (the MIC of conjugate 5 against *P. aeruginosa* and *Staphylococcus aureus* is <0.05 μg/mL and 0.4 μg/mL, respectively) [85]. Ji et al. [86] reported the synthesis and antibacterial activity of tricatechol siderophores coupled with ampicillin and amoxicillin, i.e., conjugates 6 and 7. The results showed that the activity of the two conjugates was inversely proportional to the concentration of iron, and compared with ampicillin and amoxicillin, the two conjugates acted against Gram-negative bacteria under iron deficiency. In particular, the antibacterial activity against *P. aeruginosa* was significantly improved: the MIC of conjugates 6 and 7 against *P. aeruginosa* was 0.05–0.39 μg/mL, whereas that of ampicillin and amoxicillin was >100 μg/mL. Fritsch et al. [87] synthesized MeCam(1,3,5-N,N′,N″-tris-(2,3-dihydroxybenzoyl)-triaminomethylbenzene), i.e., ampicillin conjugate 8. Although the aim was to develop a siderophore–antibiotic conjugate that inhibits *P. aeruginosa*, conjugate 8 exhibited no significant effect on *P. aeruginosa*. However, it showed strong antibacterial activity against *Escherichia coli* and *Acinetobacter baumannii*. Although the MeCam-based conjugate demonstrated no inhibitory effects on *P. aeruginosa*, the authors’ work on uncovering its uptake mechanism and response nonetheless provides a conceptual framework that paves the way for developing effective conjugates against *P. aeruginosa*. Enterocin is a natural tricatechol siderophore, which was modified by Zheng et al. [88]. Ampicillin and amoxicillin were connected to enterobacterin by stable polyethylene glycol connectors to form conjugates 9 and 10. Growth curves showed that conjugates 9 and 10 could kill *E. coli* CFT073 when the concentration was 10-fold lower than that of ampicillin and amoxicillin. The MIC of these conjugates was approximately 1000-fold lower than that of ampicillin and amoxicillin. In addition, Zheng et al. [88] also tested whether the chirality of four antimicrobial substances affected the antibacterial effect. Compared with L-isomers, the two D-type conjugates were not easily transported to various *E. coli*. These results regarding chirality are very helpful for the effective development of siderophore–antibiotic conjugates.

#### 3.1.2. Conjugates of Cephalosporin Antibiotics and Siderophores

**Figure 5:**
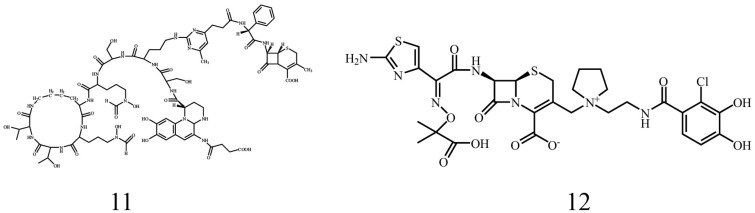
Conjugates of cephalosporin antibiotics and siderophores.

The mechanism of action of cephalosporin antibiotics is similar to that of penicillin antibiotics. Specific interference with the synthesis of bacterial cell walls causes cell death due to structural breakage. However, cephalosporin antibiotics are more stable and have a wider antibacterial spectrum than penicillin antibiotics [89]. As shown in Figure 5, Kinzel et al. purified PVD of *P. aeruginosa* ATCC15692 and prepared its conjugate 11 with Cefalexin. However, the results were unexpected, as the MIC value of conjugate 11 showed no bacteriostatic activity against *P*. *aeruginosa*. The conjugate even promoted bacterial growth, indicating that it had been ingested by bacteria, but that Cefalexin could not exert its activity due to defects in structural design [90]. Later, a new type of siderophore–cephalosporin, with a monocatechol group attached to the C3 side chain, was designed: cefiderocol (conjugate 12), which has been widely studied in recent years. Under iron deficiency, cefiderocol uptake was promoted, and antibacterial activity was enhanced, confirming the assumption that cefiderocol could inhibit bacteria via the iron transport system and monocatechol groups [91,92]. Cefiderocol is actively transported across the outer bacterial membrane into the periplasmic space via the iron transport system. Highly stable against a variety of β-lactamases, the drug accumulates continuously and thereby inhibits cell wall synthesis in Gram-negative bacteria by binding to penicillin-binding proteins [93,94]. It is worth noting that cefiderocol has undergone multiple clinical trials and has been selectively used in the treatment of Gram-negative bacterial infections, especially in patients with limited or no treatment regimens [95]. Additionally, recent reports have confirmed excellent activity of cefdil against Gram-negative bacteria, showing once again that cefiderocol is a useful complementary drug for treating resistant infections [96].

#### 3.1.3. Conjugates of Quinolone Antibiotics and Siderophores

**Figure 6:**
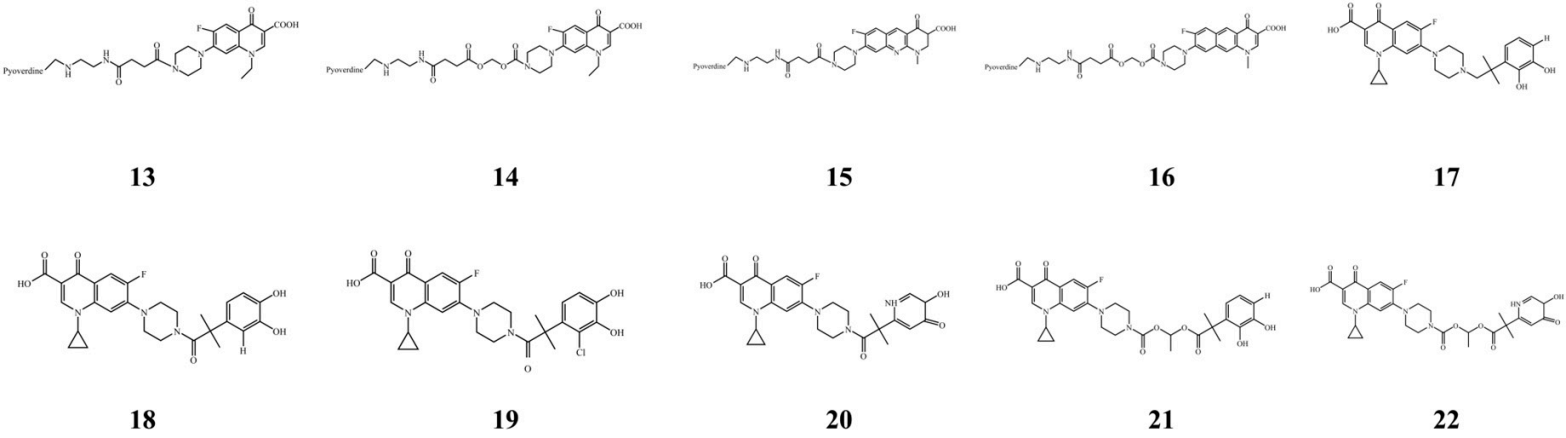
Conjugates of quinolone antibiotics and siderophores.

The outer bacterial membrane represents an obstacle for the internalization of antibiotics, especially quinolones [97]. As shown in Figure 6, Hennard et al. [98] synthesized four siderophore-based antibiotics (conjugates 13–16) formed by two quinolone-class antibacterial agents: norfloxacin (a quinolone) and benzonaphthyridone (a naphthyridone derivative). These bind to the PVD of *P. aeruginosa* ATCC 15692 through two types of linkers (one relatively stable and the other easily hydrolyzed). The antibacterial activities of these conjugates and quinolones alone were compared. Conjugating antibiotics to PVD facilitated cell internalization. In addition, when the linker is unstable and conducive to the release of antibiotics in cells, gyrase (referring to DNA gyrase) can be better inhibited, resulting in enhanced antibacterial effects. Loupias et al. [99] selected catecholates, which target two distinct classes of enzymes, i.e., penicillin-binding proteins (localized in the periplasm) and topoisomerases (localized in the cytoplasm), as the siderophore analog moiety. They synthesized six new siderophore analogues and ciprofloxacin conjugates (conjugates 17–22) against *P. aeruginosa* and *Burkholderia* spp. *(Burkholderia pseudomallei*). Among them, conjugates 17–20 were prepared by conjugating siderophore analogues with ciprofloxacin via non-cleavable linkers, and their antimicrobial activity against *P. aeruginosa* (MIC: 8–32 μg/mL) was significantly lower than that of ciprofloxacin (MIC: 1–2 μg/mL) and ceftazidime (MIC: 1–2 μg/mL). By contrast, conjugates 21–22 were synthesized by coupling siderophore analogues with ciprofloxacin through cleavable linkers. Among them, the antimicrobial activity of conjugates 21–22 against *P*. *aeruginosa* (MIC: 1–2 μg/mL) was not significantly different from that of ciprofloxacin (MIC: 1–2 μg/mL), and conjugates 21–22 exhibited excellent antibacterial activity against *P*. *aeruginosa* [100]. The mechanism underlying this result is that the cleavable linkers of conjugates 21–22 enable the effective release of ciprofloxacin inside bacterial cells after the conjugates are transported into the cells via the TonB-dependent receptor, resulting in antibacterial effects.

#### 3.1.4. Other Antibiotic–Siderophore Conjugates

**Figure 7:**
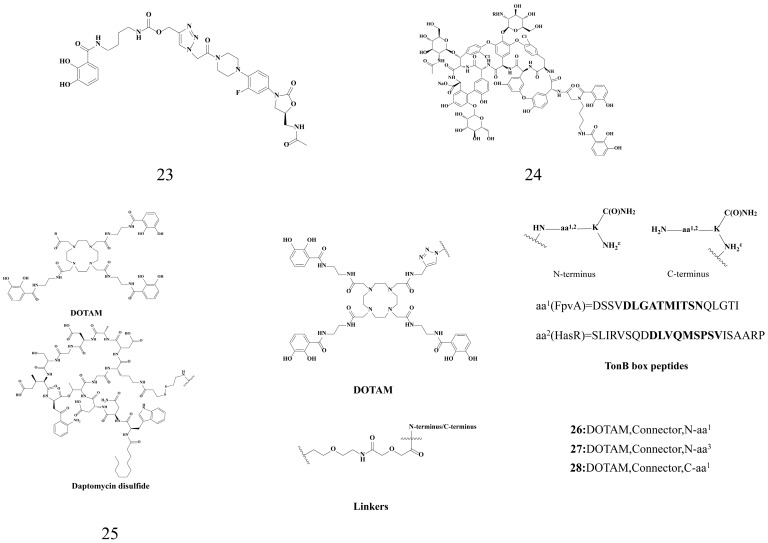
Other antibiotic–siderophore conjugates.

As shown in Figure 7, Paulen et al. constructed the conjugate 23 by coupling the oxazolidinone antibiotic linezolid with a monocatechol siderophore. This conjugate exhibits an approximately 10-fold improved MIC against *P. aeruginosa* compared with the parent antibiotic linezolid [101]. Ghosh et al. [102] recently coupled biscatechol siderophores with teicoplanin in a similar way to form conjugate 24. This conjugate retained its original activity against Gram-positive bacteria (teicoplanin only targets Gram-positive bacteria). Teicoplanin binds to peptidoglycan to inhibit bacterial cell wall synthesis. The MICs of conjugate 24 against *S. aureus* and *A. baumannii* are 0.8 μg/mL and 0.4 μg/mL, respectively, whereas the MIC of teicoplanin against *A. baumannii* is 25 μg/mL. This indicates that modifying siderophores can resolve certain limitations of antibiotics. This provides a direction for the development of siderophore conjugates, which could contribute to solving the problem of drug-resistant bacteria.

“Trojan horse” antibiotics are a broad concept, including not only typical antibiotics (e.g., ampicillin and linezolid) but also other bacteriostatic substances. Fritsch et al. [87] developed conjugate 25 by conjugating the siderophore DOTAM (1,4,7,10-tetrakis(carbamoylmethyl)-1,4,7,10-tetraazacyclododecane) with the antibiotic daptomycin. However, conjugate 25 failed to exhibit significant antibacterial efficacy against *P. aeruginosa*. Nevertheless, the investigative work on DOTAM-based siderophore conjugates has provided valuable insights and a theoretical framework for the subsequent development of targeted conjugates against *P. aeruginosa*. Peukert et al. [103] proposed a novel inhibition principle: interfering with the interaction between TonB and TonB-dependent transporters can interfere with iron homeostasis and pathogen growth, decreasing metabolism and adaptability. The authors suggest that overexpression of the TonB box peptide in the N-terminal domain of siderophore transporters can inhibit *P. aeruginosa* growth. They used covalent or cleavable linkers of varying length and attachment sites to couple TonB box peptides with synthetic DOTAM and MeCam siderophores to obtain conjugates 26, 27, and 28. The three conjugates had strong antibacterial activity against *P. aeruginosa* growth (MIC = 0.5 μM, 4 μM, and 0.1 μM, respectively).

### 3.2. PCH–Antibiotic Coupling

**Figure 8:**
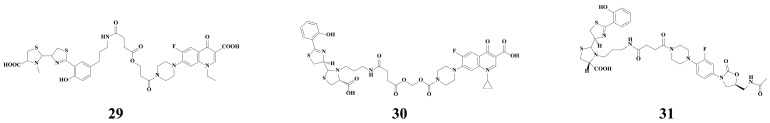
Antibiotic–pyochelin conjugates.

As early as 2007, Rivault et al. [104] modified the siderophore PCH of *P. aeruginosa*, partially functionalizing and coupling it with norfloxacin to form conjugate 29 and its derivatives (Figure 8). A stress growth curve showed that only the conjugate with an unstable connecting arm exerted bacteriostatic effects on *P. aeruginosa*; however, these effects did not exceed the lethal effect of norfloxacin on bacteria. The authors speculated that this result is due to the instability of the connecting group. It is partially hydrolyzed by non-specific enzymes when it does not reach the cytoplasm, and only a small part of the unhydrolyzed part plays a role. Conversely, the reason why other derivatives with stable connecting arms do not exert any effects is either that antibiotics cannot be effectively separated, or that the conjugates are blocked by proteins related to siderophore uptake. In the process of studying PCH and a fluorescent probe, Noel et al. [105] found that using a shorter succinic acid connecting arm or longer connecting arm helps decrease the steric hindrance of proteins related to PCH uptake and PCH derivative synthesis. This provides insights for the development of PCH conjugates. To this end, Noel et al. [106] confirmed in another experiment that N3-site-functionalized PCH (a PCH analogue) can still be bound by the outer membrane receptor FptA of PCH and transported into the cell. This suggests that this functional PCH is a good carrier for the synthesis of “Trojan horse” antibiotics. The authors coupled the quinolone antibiotic ciprofloxacin with PCH analogues to form conjugate 30, hoping to enhance the bactericidal ability of ciprofloxacin against *P. aeruginosa*. However, the results showed that the MIC of ciprofloxacin against *P. aeruginosa* was 0.04 μg/mL, which was still superior to that of conjugate 30 (0.6 μg/mL). This may be due to the insolubility of conjugate 30 in water and the instability of the connecting arm. Additionally, Paulen et al. [107] used oxazolidinone to connect with PCH and form conjugate 31. Unfortunately, the antibacterial activity of the conjugate was not significantly improved. These findings collectively highlight the core challenges in developing siderophore-based “Trojan horse” antibiotics. The stability of the linker and the solubility of the conjugate are both critical factors influencing the antibacterial activity of conjugates.

“Trojan horse” antibiotics consist of three components: siderophores (or their derivatives), linkers, and antibiotics. Each of these should first be selected and optimized separately to produce good couplings with the best biological properties in terms of membrane permeability and antibiotic activity [108]. The specific examples mentioned above suggest that more consideration should be given to the chemical properties of the linkers in the synthesis of PCH–antibiotic conjugates. This is because the conjugates that have successfully improved antibacterial activity in the past have proven the effectiveness of antibiotics. Furthermore, synthetic siderophores and derivatives (whether PVD or PCH) have also been shown to contribute to bacterial growth under iron deficiency.

## 4. Conclusions and Perspectives

Bacterial resistance has become an increasingly severe issue worldwide, evolving into a major public health challenge threatening human health [109]. *P. aeruginosa* exhibits inherent resistance to multiple antibiotics and has continuously evolved additional resistance mechanisms, such as the production of antibiotic-hydrolyzing enzymes and the formation of biofilms, during its long-term “struggle” against antibiotics [4]. These adaptations render the treatment of *P. aeruginosa* infections exceptionally challenging.

Biofilms are important virulence factors in *P. aeruginosa* and other pathogenic bacteria [110]. Therefore, exploring the effects of siderophore-mediated Trojan horse strategies on biofilm formation can expand their application scope from antibacterial activity to anti-biofilm therapy [111]. In addition, quorum-sensing inhibitors (QSIs) can downregulate the expression of virulence factors by interfering with the bacterial quorum-sensing system [112,113]. Future studies should further evaluate the regulatory effects of siderophore–QSI conjugates on the virulence of *P. aeruginosa*, thereby extending the application from antibacterial therapy to anti-virulence therapy, which is of great significance for the treatment of chronic *P. aeruginosa* infections [114]. The siderophore-mediated “Trojan horse” strategy enables targeted delivery of antibiotics by leveraging bacterial iron uptake mechanisms, offering a highly promising solution for the treatment of such refractory infections [23,115,116,117].

The design of the linker (spacer moiety) is a key factor influencing the antibacterial activity of the conjugate, with its length and stability being critical [98]. An excessively large linker increases the steric hindrance of the conjugate, impeding its penetration through the bacterial cell membrane and thus preventing its internalization into the cell in which it can exert antibacterial effects. Conversely, premature cleavage of the linker before the drug reaches the cytoplasm causes the antibiotic to dissociate from the siderophore prematurely. This prevents effective delivery to the target site via the iron uptake system, preventing bacteriostatic activity [99].

Furthermore, the stability of siderophore–antibiotic conjugates is influenced by several key physicochemical factors. Among these, environmental pH has the most significant impact: ester, amide, or hydrazone bonds between the antibiotic and siderophore are susceptible to hydrolysis under strongly acidic or alkaline conditions, leading to premature dissociation [118]. Temperature also plays an important role, as elevated temperatures accelerate bond cleavage and molecular degradation [119]. Light exposure is particularly relevant for quinolone-containing conjugates (e.g., norfloxacin and benzonaphthyridone derivatives), as quinolones are prone to photodegradation, which may cause phototoxicity [120]. Understanding these factors is essential for the rational design of stable and effective siderophore–antibiotic conjugates, as well as for determining appropriate storage and handling conditions.

The antibiotic (active moiety) is the core component responsible for the conjugate’s antibacterial effects. Once inside the bacterial cell, the antibiotic inhibits the activity of key targets, interferes with normal bacterial growth and metabolic processes, and ultimately suppresses growth or induces bacterial death. With advances in antibacterial drug research, the conjugation of antimicrobial peptides and other antibacterial molecules with siderophores has opened new avenues for the development of the “Trojan horse” strategy [23]. Antimicrobial peptides, also known as host defense peptides, are small-molecular-weight peptides with antibacterial activity and serve as important components of the innate immune system [121]. They not only exhibit bactericidal and fungicidal activity but also play crucial roles in immune regulation and wound healing stimulation [122]. Studies have shown that antimicrobial peptides exert antibacterial effects through multiple mechanisms, such as enhancing membrane permeability (thereby promoting cell membrane lysis and cell content release) and inhibiting protein synthesis [123]. Conjugating antimicrobial peptides with siderophores is expected to enable efficient delivery of antimicrobial peptides into bacterial cells via iron uptake systems, maximizing their unique antibacterial mechanisms and providing a novel approach for treating drug-resistant bacterial infections.

Beyond antimicrobial peptides, several natural products and small-molecule compounds with antibacterial activity also have potential for conjugation with siderophores [124]. For example, flavonoids and terpenoids isolated from plant extracts exhibit antibacterial properties [125], and their conjugation with siderophores is anticipated to overcome their delivery limitations during antibacterial therapy, thereby enhancing their efficacy. To facilitate the design and optimization of such siderophore conjugates, researchers have increasingly leveraged rapid advancements in computational biology and bioinformatics. As a result, in silico technologies have been extensively applied to predict intermolecular interactions, screen potential active compounds, and discover anti-biofilm agents [126]. Specifically, molecular docking and virtual screening can predict the binding affinity of conjugates with outer membrane receptors, while machine learning models can optimize the chemical structure of linkers and predict the stability and antibacterial activity of siderophore–antibiotic conjugates [127,128,129]. Furthermore, bioinformatics analysis of siderophore biosynthetic gene clusters can also provide a theoretical basis for screening and designing suitable siderophore analogs. Together, these computational approaches enable the development of novel conjugate types, which can greatly expand the applicative scope of the “Trojan horse” strategy for treating infections, thereby paving the way for the creation of new antibacterial drugs.

The “Trojan horse” strategy offers a promising avenue for addressing *P. aeruginosa* resistance. By skillfully conjugating antibiotics with siderophores, it achieves efficient drug delivery and has demonstrated significant antibacterial effects in preclinical studies and clinical trials. With ongoing in-depth research and expansion, the development of novel conjugates will further enrich anti-infection therapeutic options, providing additional solutions to combat the growing crisis of drug-resistant bacterial infections.

## Data Availability

No new data were created or analyzed in this study. Data sharing is not applicable to this article.

## Abbreviations

The following abbreviations are used in this manuscript:

Dha	Dehydroalanine
DOTAM	1,4,7,10-tetrakis(carbamoylmethyl)-1,4,7,10-tetraazacyclododecane
L-Dab	L-2,4-diaminobutyrate
L-fOHOrn	L-N_5_-formyl-N_5_-hydroxyornithine
MeCam	1,3,5-*N*,*N*′,*N*″-tris-(2,3-dihydroxybenzoyl)-triaminomethylbenzene
MIC	Minimum inhibitory concentration
NRPS	Non-ribosomal peptide synthetase
NTN	N-terminal nucleophile
PCH	Pyochelin
PVD	Pyoverdine
PVDI	Pyoverdine type I
PVDII	Pyoverdine type II
PVDs	Pyoverdines (types I–IV)
QSIs	Quorum-sensing inhibitors
